# Transportation model utilized in the first week following the Kahramanmaraş earthquakes in Turkey - transport health centers

**DOI:** 10.1186/s13049-023-01108-7

**Published:** 2023-08-22

**Authors:** Sarper Yilmaz

**Affiliations:** Department of Emergency Medicine, Kartal Dr. Lütfi Kırdar City Hospital, Istanbul, Turkey

On February 6, 2023, a formidable earthquake registering 7.7 on the Richter scale struck the Pazarcık district of Kahramanmaraş province. Mere nine hours later, a second earthquake with a magnitude of 7.6 shook the same region, resulting in a substantial loss of life and extensive destruction. The impact of these twin earthquakes was severe, prompting the declaration of a state of emergency in ten affected provinces. Furthermore, the World Health Organization (WHO) classified the situation as a Level 3 Emergency [1]. According to the report from the Turkish Emergency Medicine Association, the earthquake’s impact extended to ten provinces, namely: Kahramanmaraş, Hatay, Gaziantep, Osmaniye, Malatya, Adana, Diyarbakır, Şanlıurfa, Adıyaman, and Kilis. Official figures reveal that the earthquakes claimed 50,399 lives, left 80,278 injured, and led to the collapse of 6,444 buildings. The direct impact affected a staggering 13.5 million people in Turkey, resulting in 850 limb amputations. Remarkably, the aftershocks continued with a total of 9,990 recorded in the second month following the Kahramanmaraş earthquakes [1, 2].

According to the Field Observation Report from the Disaster Commission of the Emergency Medicine Association of Turkey (EMAT): In the regions affected by the earthquake, there was a notable upsurge in patient admissions, particularly in the immediate aftermath of the seismic event [2]. As a consequence of the earthquake, most hospitals were compelled to function solely on their ground floors until field hospitals could be established in the subsequent days. Evacuations were necessary for a significant number of patients in regular wards and intensive care units. While certain hospitals were rendered inoperative due to the earthquake, others persevered in their operations despite sustaining damage. The persistent aftershocks triggered a disaster response in 10 provinces, leading to substantial damage and incapacitation of numerous hospitals. Both primary and advanced medical treatments (including surgeries and internal procedures) reached a point where health institutions and healthcare personnel found them insufficient to cope with the escalating demands.

The earthquake resulted in significant damages, including widespread impact on health facilities in ten provinces. Turkey devised two strategies to address the extensive devastation. The first approach involved dispatching both national and international health aid, comprising healthcare workers and essential equipment, to the affected provinces. Additionally, injured individuals were transferred from the damaged regions to healthcare facilities located outside the disaster area. However, due to the large size and high population density of the earthquake-affected zone, Turkey had to implement three distinct transportation measures: transportation of healthcare workers, evacuation and transfers of injured individuals, and transportation of essential medical equipment (Table 1).

**Table 1:** Transportation strategy used in the earthquakes in Kaharamanmaraş, Turkey

	Method	Management
A. Transportation of HCWs	Roadway	Alternative routes were created on blocked roads.
	Air	Airports in major cities (Istanbul, Ankara, Izmir) have been transformed into disaster centers to transport health and rescue care workers to disaster areas.
B. Evacuation and Transfers	Roadway	Transfer to nearby cities in the disaster zone
	Air	Transfers to distant cities (Istanbul, Ankara, Izmir)
	By Sea	Transfer to nearby cities (Mersin)
C. Transportation of Equipment	Roadway	Inventory allocation from different locations
	Air	Utilization of central storages

HCWs: health care workers.

1. **Step: Providing transportation for health care workers**.
   The earthquake occurred on February 6, 2023, at 04:17. In the immediate aftermath of the seismic event, the advance medical response teams (UMKE - National Medical Search and Rescue) promptly mobilized via roadways. Despite the establishment of multiple routes, accessing the earthquake-affected regions proved challenging through most of the road network. Several factors contributed to disruptions in road traffic in the days following the earthquake: 1) The damage inflicted by the seismic activity on the road infrastructure, 2) The snowy and cold weather conditions typical of February, and 3) Efforts by civilians residing outside the disaster zone to reach their affected relatives using private vehicles. Health personnel faced the necessity of frequently altering their road routes to reach the disaster-stricken areas. Furthermore, the earthquake caused damage to numerous roads, overpasses, and underpasses, while the harsh winter conditions also impeded transportation on unaffected roadways. To facilitate the timely access of health workers to the earthquake-affected areas, authorities adopted an early strategy that involved employing military and civilian air transport options.
2. **Step: Transportation of medical equipment**.
   The main mode of transportation for medical equipment and supplies to the earthquake-affected regions was via road, complemented by the use of air transportation support. The introduction of personnel transportation to the disaster-stricken areas through air travel not only eased the transportation of medical supplies by road but also played a pivotal role in the timely procurement of essential materials for disaster relief.
3. **Step: Evacuation and transfers of injured patients**.
   Turkey had to devise an unconventional transportation approach, rarely encountered in the disaster literature. To manage the influx of earthquake-affected patients from seven severely damaged provinces, three major cities (Adana, Mersin, and Diyarbakir), which experienced lesser damage in the disaster, were transformed into Transport Health Centers. These patients were transported to these centers by land, air, and sea and subsequently treated in hospitals resembling field tents within the Transport Health Centers. During the initial stages, patients received primary treatment and were airlifted to distant provinces. According to officials, this strategic approach facilitated the transfer of a total of 1715 patients to hospitals in various cities via airplanes and helicopters in response to the earthquakes centered in Kahramanmaraş. (Fig. 1) [3].
  **Fig. 1:**
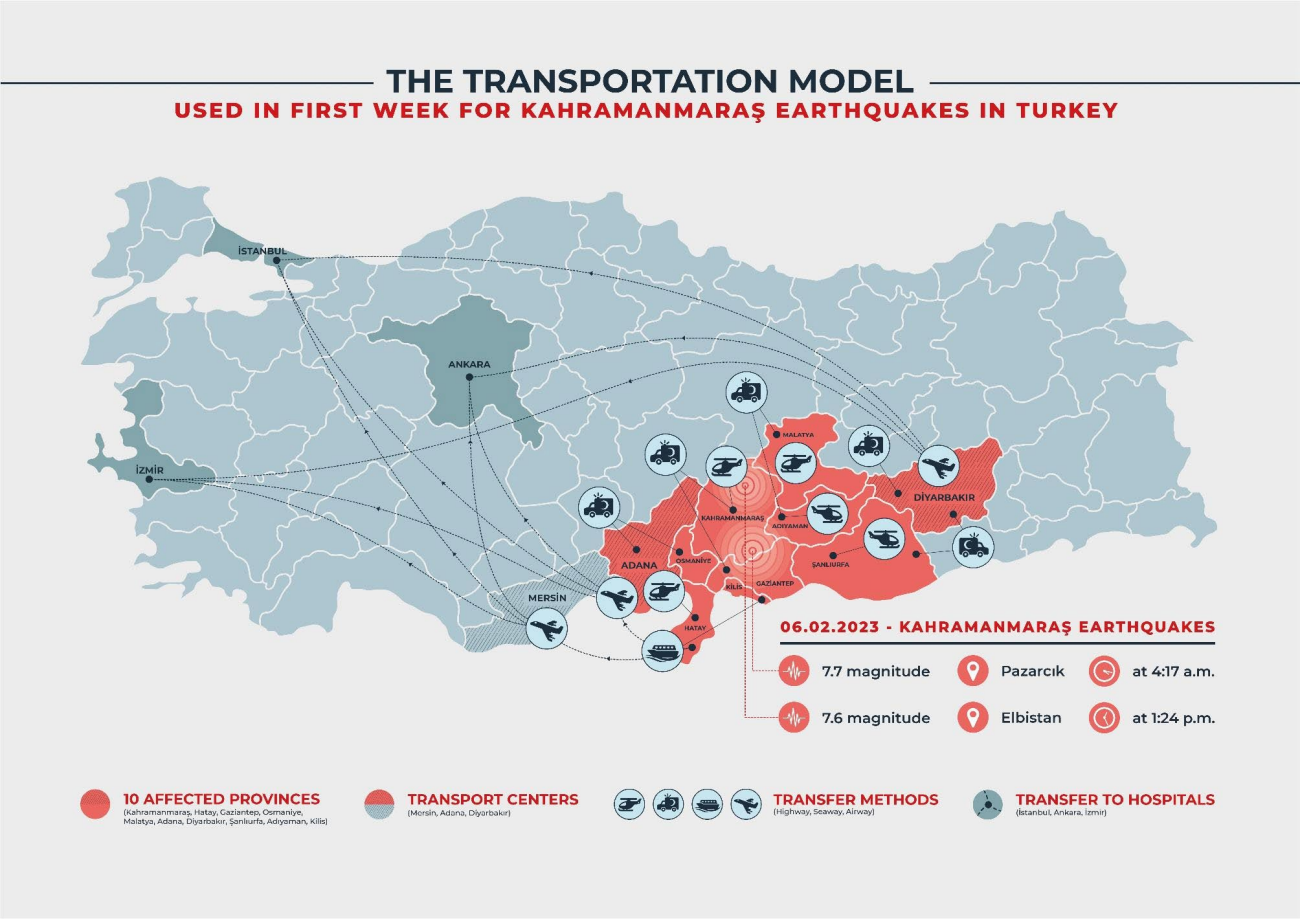
Transportation Hubs and Routes for Earthquake-Affected Regions in Kahramanmaraş, Turkey

In the event of a significant disaster, the implementation and advancement of “Transport Health Centers” as a transportation model should be both utilized and further developed. The invaluable lessons gleaned from past disasters enable societies to enhance their preparedness for future calamities, encompassing both earthquake-related disasters, actions to be undertaken during seismic events, and the streamlining of healthcare service delivery in the post-earthquake aftermath [2, 4]. Consequently, it is crucial to scrutinize and explore the continued evolution of this approach, considering its potential application in handling mass casualty incidents and other natural disasters. In light of the escalating number of mass casualties globally, a comprehensive examination and refinement of this transportation model are imperative to effectively address the challenges posed by such incidents.

## Data Availability

Not applicable.
