# Correction: Transportation model utilized in the first week following the Kahramanmaraş earthquakes in Turkey - transport health centers

**DOI:** 10.1186/s13049-023-01136-3

**Published:** 2023-10-31

**Authors:** Sarper Yilmaz

**Affiliations:** Department of Emergency Medicine, Kartal Dr. Lütfi Kırdar City Hospital, Istanbul, Turkey

**Correction: Scandinavian Journal of Trauma, Resuscitation and Emergency Medicine (2023) 31:40**

10.1186/s13049-023-01108-7

The Reference list of the original article [1] unfortunately included an incorrect reference 1:

The correct reference should read: Tao W, Jie C, Yujiang Z, et al. Preliminary investigation of building damage in Hatay under February 6, 2023 Turkey earthquakes. Earthq Eng Eng Vib. Published online July 4, 2023. doi:10.1007/s11803-023-2201-0.

The original article has already been revised.
